# Inter-Brain Synchronization during Social Interaction

**DOI:** 10.1371/journal.pone.0012166

**Published:** 2010-08-17

**Authors:** Guillaume Dumas, Jacqueline Nadel, Robert Soussignan, Jacques Martinerie, Line Garnero

**Affiliations:** 1 Université Pierre et Marie Curie-Paris 6, Centre de Recherche de l'Institut du Cerveau et de la Moelle épinière, UMR-S975, Paris, France; 2 Inserm, U975, Paris, France; 3 CNRS, UMR 7225, Paris, France; 4 CNRS, USR 3246, Centre émotion, Pavillon Clérambault, Hôpital de La Salpêtrière, Paris, France; 5 Centre des Sciences du Goût et de l'Alimentation, UMR-6265 CNRS, 1324 INRA, U-B, Dijon, France; Kyushu University, Japan

## Abstract

During social interaction, both participants are continuously active, each modifying their own actions in response to the continuously changing actions of the partner. This continuous mutual adaptation results in interactional synchrony to which both members contribute. Freely exchanging the role of imitator and model is a well-framed example of interactional synchrony resulting from a mutual behavioral negotiation. How the participants' brain activity underlies this process is currently a question that hyperscanning recordings allow us to explore. In particular, it remains largely unknown to what extent oscillatory synchronization could emerge between two brains during social interaction. To explore this issue, 18 participants paired as 9 dyads were recorded with dual-video and dual-EEG setups while they were engaged in spontaneous imitation of hand movements. We measured interactional synchrony and the turn-taking between model and imitator. We discovered by the use of nonlinear techniques that states of interactional synchrony correlate with the emergence of an interbrain synchronizing network in the alpha-mu band between the right centroparietal regions. These regions have been suggested to play a pivotal role in social interaction. Here, they acted symmetrically as key functional hubs in the interindividual brainweb. Additionally, neural synchronization became asymmetrical in the higher frequency bands possibly reflecting a top-down modulation of the roles of model and imitator in the ongoing interaction.

## Introduction

From a traditional information-processing perspective, communication is said to occur when messages flow from one location to another and cause a change in the receiver [1]. In this emit/receive/answer telegraphist model of communication, the actions of the partners are taken to be discrete signals. A more appropriate model of human communication, however, consists in considering both synchronic and diachronic aspects of communication to be entwined [2]. Indeed, during communication, both participants are continuously active, each modifying their own actions in response to the continuously changing actions of their partner. This continuous mutual adaptation generates synchrony [3] and turn-taking [4]–[6] between partners, resulting in interactional synchrony.

Taking seriously the neural exploration of communication is challenging in two ways. The first challenge is to design a suitable procedure for the study of interactional synchrony. So-called interactive paradigms mainly consist in non contingent social stimuli that do not allow true social interaction [7]. Our choice was to delineate an imitative procedure allowing synchrony and turn-taking to spontaneously take place. In effect, during an imitative interaction, each partner alternately initiates or imitates actions and both coregulate the synchronous matching [8], [9]. As a paradigm, imitative interaction offers the double advantage of delineating brain areas of interest already informed by previous research on imitation, and of recording new data concerning spontaneous interactional synchrony.

Recording interactional synchrony in an attempt to elucidate the interindividual neural mechanisms of human interaction remains an open challenge, as is the objective of moving toward two-person neuroscience [10]. Until now indeed, most fMRI explorations of interpersonal processes have scanned one individual only [11], [12] or several individuals separately in front of the same visual scene [13].

Simultaneous fMRI or EEG recordings of several brains (i.e. hyperscanning) have recently opened a new field [14]–[17]. This new field, however, has revealed rapidly how difficult it is to ‘let humans interact socially while probing their brain activity’, as said by Montague and colleagues [18]. Using dual-EEG recordings, Tognoli, Lagarde, DeGuzman and Kelso [19] asked pairs of participants to execute self-paced rhythmic finger movements with and without vision of each other. Episodes with vision generated in-phase and anti-phase motor coordination. A neuromarker of social coordination (called the phi complex) was detected over the right centroparietal area in the 9.2–11.5 Hz range for each subject of the pair separately, but interbrain synchronization of social coordination was not directly tested. Lindenberger and colleagues [20] actually explored interbrain dynamics as they found phase synchronization in the theta frequency range between frontal areas of pairs of guitarists coordinated via a metronome. However they did not reach social interaction since the coordination was obtained via an external medium. More recently, Astolfi and colleagues [15] achieved the challenge to estimate functional interbrain connectivity related to decision making in a card game task during EEG hyperscanning recording. Only the players belonging to the same team across the different tables showed significant functional connectivity between the estimated cortical signals in the α, β and γ frequency bands, with a causal relation appearing between the prefrontal area 8 and 9/46 of the first player and the anterior cingulate cortex and parietal areas of the second player. It was suggested that this causal relation may reflect cooperation between individuals, at least when decision making is related to an anticipation of the other's intention.

In the present study, we scanned pairs of subjects imitating each other at will. Though imitation is commonly considered as a foundation for learning, socialization and communication [21], [22], its use as a paradigm has been limited so far to test the direct matching hypothesis in an intraindividual perspective [23]–[27]. Here imitation was used in an interpersonal context with the aim to contribute identifying neurodynamic signatures of human interactions.

Adapted to the new challenge of understanding how neural networks exchange information [28], [29], neurodynamic tools provided by nonlinear methods [28], [30] allow measuring neural synchronizations between distant brain regions of interacting individuals. We hypothesized interbrain synchronization in parietal and frontal regions, based on intraindividual fMRI results in imitation of hand movements [27]. We expected phase synchronization of the right parietal cortices of the two partners given the pivotal role attributed to the right temporoparietal junction in social interaction [31], self-other discrimination and perspective taking [31]–[33]. Following the suggestion that multi frequency synchrony is a signature of integrative brain processes [28], [34], we expected a distributed pattern of interbrain oscillatory couplings when the interacting dyads are engaged in synchronous hand movements with turn-taking between model and imitator.

## Methods

### Ethics statement

Experiments were approved by the Ethical Committee for Biomedical Research of Pitié-Salpêtrière Hospital in Paris (agreement #07024). Participants had given their written informed consent according to the declaration of Helsinki and were paid for their participation to the study.

### Participants

Twenty two healthy young adults (5 female-female and 6 male-male pairs) of mean age 24.5 years (*SD* = 2.8) participated in the study. They were all right-handed and had normal or corrected-to-normal vision. None of them reported a history of psychiatric or neurological disease.

### Dual behavioral data acquisition

**Figure 1:**
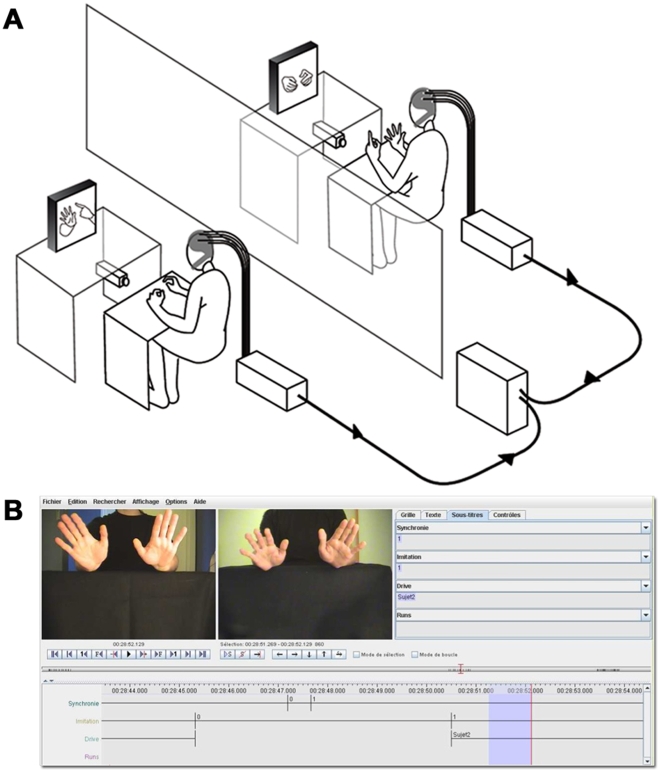
Experimental design and coding software. A. Apparatus and experimental setting of the double video system and dual-EEG recording. B. ELAN software window during an indexing session.

The experiment was conducted in three connected laboratory rooms, one for each participant and the third one for the computerized monitoring of the experiment. The participants were comfortably seated, their forearms resting on a small table in order to prevent arms and neck movements. They were told that they will have to move their hands with meaningless gestures and will watch a library of meaningless movements that will give them some examples. They could see their partner's hands through a 21-in. TV screen. Two synchronized digital video cameras filmed the hand movements. The set-up was similar to the double-video system designed by Nadel and colleagues for their developmental studies of sensitivity to social contingency in infants [9], [35], [36], except that a dual-EEG recording system was added (see Figure 1A). The session start was signaled by a LED light controlled manually, via a switch, by an experimenter located in the recording room. The output of the video records was transmitted to two TV monitors installed in the recording room allowing the experimenter to control that participants followed the requested instructions.

### Dual-EEG data acquisition

Neuroelectric activity in both participants of each dyad was simultaneously and continuously recorded at a time scale enabling to compare the EEG activity among four frequency bands: theta (4Hz–7Hz), alpha-mu (8–12 Hz), beta (13–30 Hz) and gamma (31–48 Hz). The system was composed of two Acticap helmets with 32 active electrodes arranged according to the international 10/20 system. We modified the helmets in order to cover at best the occipito-parietal regions. Four electrodes T7, T8, CP9 and CP10 were rejected due to artifacts. Ground electrode was placed on the right shoulder of the participants and the reference was fixed on the nasion. The impedances were maintained below 10kΩ. Data acquisition was performed using a 64-channels Brainamp MR amplifier from the Brain Products Company (Germany). Signals were analog filtered between 0.16Hz and 250Hz, amplified and digitalized at 500Hz with a 16-bit vertical resolution in the range of +/−3.2 mV. Note that both subjects were connected to the same amplifier that guaranteed millisecond-range synchrony between the two EEG recordings.

### Protocol

The experimental protocol (See Table 1) was divided into two blocks separated by a 10 min rest. Each block comprised four runs of 1 min 30 sec. A run was composed of three conditions: a joint observation of a prerecorded Library of 20 Intransitive (meaningless) Hand Movements (LIHM), a Spontaneous Imitation episode where the partners were told to imitate whenever they would like it (SI), and an episode where one of the partner was told to imitate the other (Induced Imitation: II) while the other was asked to move hands, with a counterbalanced order in block 2. Each run started by a 15 sec ‘No View (blank screen) No Motion (NVNM) baseline. For SI and II conditions, a 15 sec ‘No View Motion’ (NVM) baseline followed where the participants were asked to move their hands with meaningless gestures.

**Table 1:** Experimental schedule.

**Condition(Block 1)**	NVNM+Library of Intransitive Movements (LIHM)	NVNM+NVM+Spontaneous Imitation (SI)	NVNM+NVM+Induced Imitation (II) Subject A: imitator Subject B: model	NVNM+NVM+Induced Imitation (II) Subject B: imitator Subject A: model
**Pause: 10 min**				
**Condition (Block 2)**	NVNM+Library of Intransitive Movements (LIHM)	NVNM+NVM+Spontaneous Imitation (SI)	NVNM+NVM+Induced Imitation (II) Subject B: imitator Subject A: model	NVNM+NVM+Induced Imitation (II) Subject A: imitator Subject B: model
**Duration**	15s+1min 30s	15s+15s+1min 30s	15s+15s+1min 30s	15s+15s+1min 30s

### Behavioral data analysis

The video records of hand movements during the free episodes of imitation of each other's hand movements were digitized. Then, the LED signals recorded on the two video at the beginning of each session was used to synchronize the frames of the two partners. They were coded using a revised version of the ELAN program [37], [38] that offers a simultaneous presentation of two frames from different sources on the ELAN window. This software allows an analysis of the behavioral frames on separate channels of the window and a recording of time (latency, duration) and occurrence of behavioral events. This way, two main events were analyzed in each run of SI for the two partners: imitation and synchrony of hand movements.

Synchrony was assessed when the hands of the two participants started and ended a movement simultaneously, thus showing a coordinated rhythm. The criterion of simultaneity used was the co-occurrence of two gestural movements within the same video frame. This rhythm could include similar or different movements. We labeled respectively *Sync* and *NSync* the periods with and without synchrony. Imitation was assessed when the hand movements of the two partners showed a similar morphology (describing a circle, waving, swinging …) and a similar direction (up, down, right, left…). We labeled respectively *Im* and *NIm* the periods with imitation and without imitation. For each imitative episode, the individual who started a hand movement followed by the partner was labeled *the model*, and the follower was labeled *the imitator*.

The reliability of our fine grained analysis was assessed using Cohen's kappa. Inter-observer agreement between two independent coders was performed on 25% of the recordings. The values of kappa coefficients were 0.83 for imitation, 0.91 for synchrony, and 0.82 for the roles of model and imitator.

The number of switches between the model and the imitator was also computed for the SI condition, thus providing information concerning turn-taking. Finally we computed the degree of symmetry of roles within each pair of dyads, using the formula:
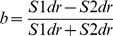
(1)where S1dr and S2dr represent the time spent as model by subject 1 and subject 2 respectively in the SI condition, b = 0 indicating a perfect symmetry of the two roles.

### EEG artifacts

The correction of eye blink artifacts in the EEG data was performed using a classical PCA filtering algorithm [39]. All the computations mentioned here and afterwards were performed within the Matlab environment. We used 800ms windows with 400ms of overlap. For each window, a principal component analysis (PCA) was performed on the raw signal and all the components were compared to an estimation of the electrooculogram (EOG) from the difference between the mean of the raw channels FP1 and FP2 and the nasion reference. If the correlation between the reconstructed EOG signal and each component of the PCA exceeded an adaptive threshold, the eigen value related to the component was fixed to zero. Then the converted EEG signal was reconstructed by using the inverse solution of the PCA. The adaptive threshold was proportional to the standard deviation of the considered i^th^ component divided by those of the current window signal:
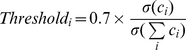
(2)where 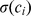 stands for the standard deviation of the i^th^ component of the PCA and 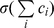 is the standard deviation of the signal. EEG signals were then visually checked to exclude muscular artifacts from the analysis. A Hamming window was used to control for artifacts resulting from data splicing.

### EEG neurodynamic analysis

EEG data during SI and II conditions were analyzed using the phase locking value (PLV) in order to detect adjustment of rhythmicity between two distant brain recordings. Following filtering corrections, EEG data were re-referenced to a common average reference (CAR) and transformed by discrete Hilbert methods for specific narrow frequency bands: theta (4–7Hz), alpha-mu (8–12Hz), beta (13–30Hz) and gamma (31–48Hz). Phases and amplitudes extracted using the Hilbert transform on all band passed signals met the reliability criteria defined in past studies [40]. For the SI condition, the EEG data were segmented into 800ms windows and mapped with the corresponding behavioral samples of synchrony (*Sync*), no synchrony (*NSync*), imitation (*Im*), and no imitation (*NIm*).

The interbrain analysis was done with the PLV for each pair (i,k) of electrodes between the two helmets (electrode i and k respectively for the helmets 1 and 2). This was done for each frequency band according to the relation:
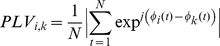
(3)where N is the number of samples considered in each 800ms window, 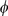 is the phase and | | the complex modulus. Thus, PLV measure equates 1 if the two signals are perfectly phase locked across the whole time window observed, and equates 0 if they are totally unsynchronized. Thus, PLV is equal to one minus the circular variance of phases' differences.

### Statistical analysis

For the SI condition, nonparametric methods were used to compare phase synchronization of oscillatory activity during 800ms epochs for synchronous versus non-synchronous acts (*Sync vs. NSync*), and for imitative versus non imitative acts (*Im vs. NIm*). The II condition and the No View Motion baseline condition were compared (*II vs. NVM*) similarly. The high dimension of the PLV spaces leads to create an extension of the classic clustering algorithm adapted to the hyperscanning. Notably, t-values were first computed for the PLVs related to all electrodes of helmet 1 paired one by one with all electrodes of helmet 2. Following previous studies [41], [42], the resulting t-value matrices were then thresholded for absolute values larger than 2. Selected pairs of electrodes were then clustered according to a neighborhood criterion adapted to PLVs between two helmets. Pairs of electrodes between two EEG helmets are couple of electrodes formed by one electrode on one helmet with one electrode on the other helmet. Two pairs of electrodes on two helmets were considered neighbors if the two electrodes on the same helmet were neighbors. Two pairs of electrodes can also share a common electrode on the same helmet. In this case, the other extremities of the pairs have to be neighbors. Thus, three cases of neighborhood can be found:

- two side-by-side electrodes on the helmet of subject 1 connected respectively to two side-by-side electrodes on the helmet of subject 2.
- one electrode on the helmet of subject 1 connected with two side-by-side electrodes on the helmet of subject 2.
- one electrode on the helmet of subject 2 connected with two side-by-side electrodes on the helmet of subject 1.

We took as the cluster statistics the sum of all t-values of the pairs members of the cluster. We performed multiple comparisons procedures by doing bootstraps on the cluster statistics [43]. The thresholds that control the family wise error rate (FWER), representing the probability of making false discoveries, were determined by non parametrical permutation methods. Statistics were corrected through both spatial (pairs of electrodes) and spectral dimensions (frequency bands) by taking the maximal t-value for each permutation. All randomizations were done for a rejection of the null hypothesis and a control of false alarm rate at *p = 0.05*.

## Results

### Behavioral data

#### Symmetry of roles of imitator and model

Symmetry of roles of model and imitator within each pair of subjects was computed, a value of 0 indicating an ideal balance between the two roles within a dyad. Two dyads of subjects were excluded from further analyses as they exceeded 3 standard deviations from mean index of symmetry. The mean value across the remaining 9 dyads was close to 0 (M = 0.02 SD = 0.14), thus revealing a good turn-taking of roles.

#### Dyadic episodes of imitation and synchrony for SI

The proportion of time spent imitating the partner's hand movements, exhibiting interactional synchrony and imitating synchronically the partner's hand movements, was measured in all runs for SI condition (See Table 2). The participants were preferentially involved in imitation (M = 64.69% of the interaction time) rather than in moving their hands independently, and were most often synchronized (M = 78% of the time).

**Table 2:** Mean (and SD) percent time spent synchronizing and/or imitating hand movement during spontaneous imitation condition.

	Imitation		Non-Imitation		Total	
	*M*	*SD*	*M*	*SD*	*M*	*SD*
**Synchrony**	51.27%	16.59%	26.66%	12.77%	77.93%	17.63%
**Non-Synchrony**	13.42%	13.62%	08.65%	05.56%	22.07%	17.63%
**Total**	64.69%	13.74%	35.31%	13.74%		

### Neurodynamic results

Using fine grained video coding of behavioral parameters in the SI condition, we compared EEG contrasts between synchronized versus non-synchronized episodes and between imitative versus non imitative episodes.

#### Synchronized versus non-synchronized episodes of SI condition

**Figure 2:**
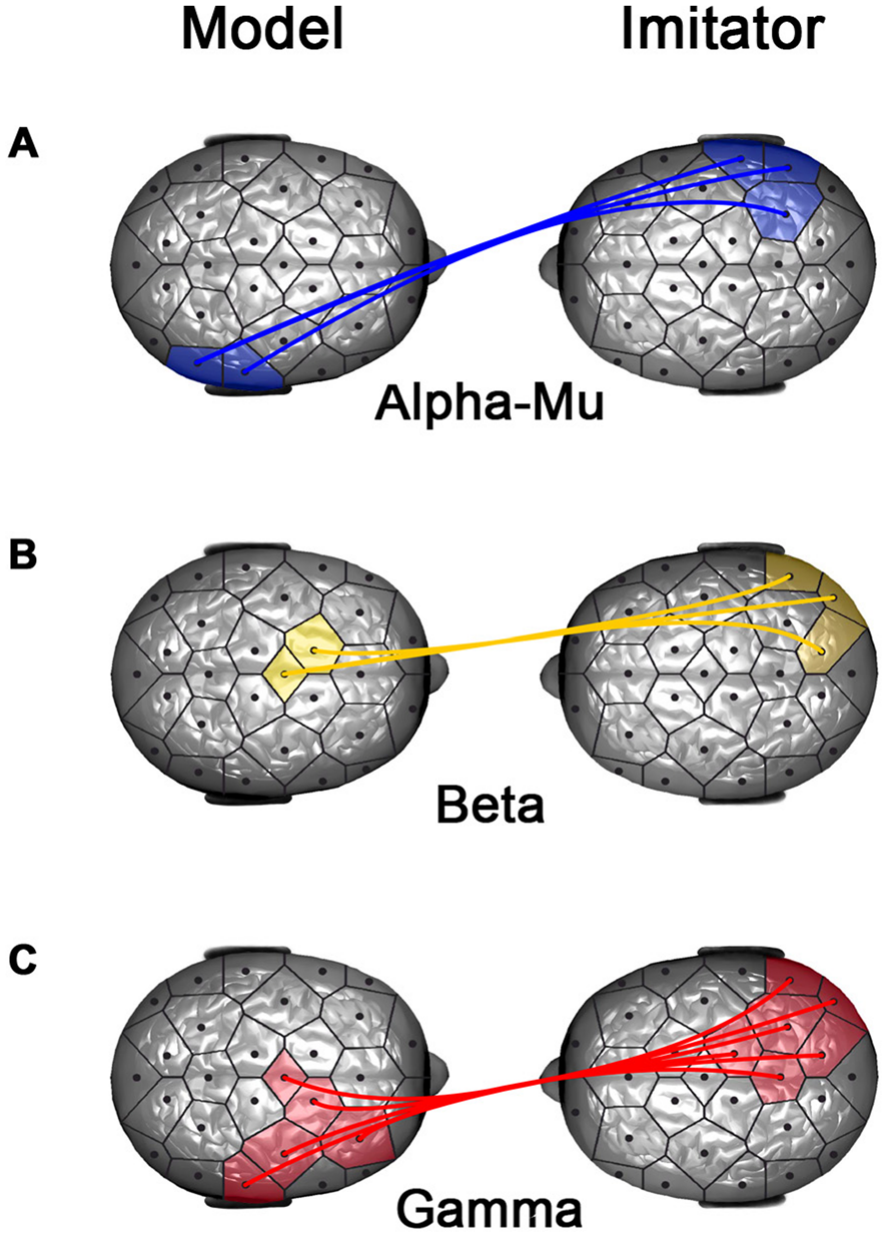
Intersubject neural synchronizations during interactional synchrony. Representation of statistically significant (P<0.05, nonparametric permutation test, corrected for multiple comparisons) coupling (PLV) for all subjects between electrodes of the model and the imitator: comparison for spontaneous imitation trials between behavioral synchrony episodes and those with no behavioral synchrony (Sync vs. NSync). On the left of the figures the participants are models, on the right the participants are imitators. A. Alpha-Mu band cluster between right centro-parietal regions. B. Beta band cluster between central and right parieto-occipital regions. C. Gamma band cluster between centroparietal and parieto-occipital regions.

Significant EEG contrasts were found between Sync and NSync episodes (which mostly included imitation). Figure 2 depicts the interbrain dynamical networks of phase synchronization among alpha-mu, beta and gamma frequency bands. The cluster statistics (CS) provide inter-frequency comparisons with the following absolute thresholds: 6.3, 7.8, 8.9 and 11.5 for respectively p<*0.05*, *p<0.01*, p<*0.005* and p<*0.001*.

**Figure 3:**
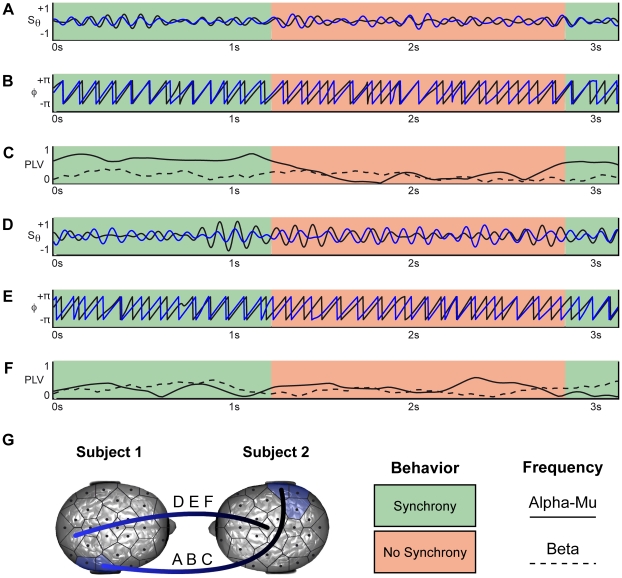
Brain synchronization: online example. Samples of spontaneous imitation episodes in the dyad n°3 showing the correspondence between interactional synchrony and brain activities. The green areas indicate periods where subjects were behaviorally synchronized and the red ones periods without behavioral synchrony. A. Time course of normalized EEG signal filtered in the alpha-mu frequency band for the channels P8 of both subjects. These channels are members of the cluster shown in figure 2A. B. Phase extracted from the signals. C. PLV calculated with sliding centred time windows of 800ms length in the alpha-mu band (related to A and B) quantifying the neural synchronization between the two subjects. Beta band PLV for the same electrodes is also shown in dashed line. D. Time course of normalized EEG signal filtered in alpha-mu frequency band for the channels PO2 in Subject 1 and Cz in Subject 2. Those channels are not members of any clusters. E. Phase extracted from the signals. F. PLV calculated with sliding centred time windows of 800ms length in the alpha-mu band (related to D and E) quantifying the neural synchronization between the two subjects. Beta band PLV for the same electrodes is also shown in dashed line. G Representation of the pairs of electrodes P8-P8 (A,B,C) and PO2-Cz (D,E,F).

Symmetrical increase in PLV was found between the right parietal regions of the model (CP6, P8) and of the imitator (CP6, P4, P8) in the alpha-mu frequency band (see Figure 2A with CS = +6,7, p<*0.05*). The central region (FC1, Cz) of the model's brain and the parieto-occipital brain region (P8, PO2, PO10) of the imitator were synchronized in the beta frequency band (see Figure 2B; with CS = +6.4, p<*0.05*). Finally, a wide frontal central area (F4, FC2, Czar, C4, CP6) of the model's brain was synchronized with the parietal area (CP2, PZ, P4, P8, PO2, PO10) of the imitator's brain for the gamma frequency band (see Figure 2C, with CS = +17.4, p<*0.001*). As an example, Figure 3 illustrates phase synchronizations between brains in a dyad during episodes of spontaneous imitative exchanges.

**Figure 4:**
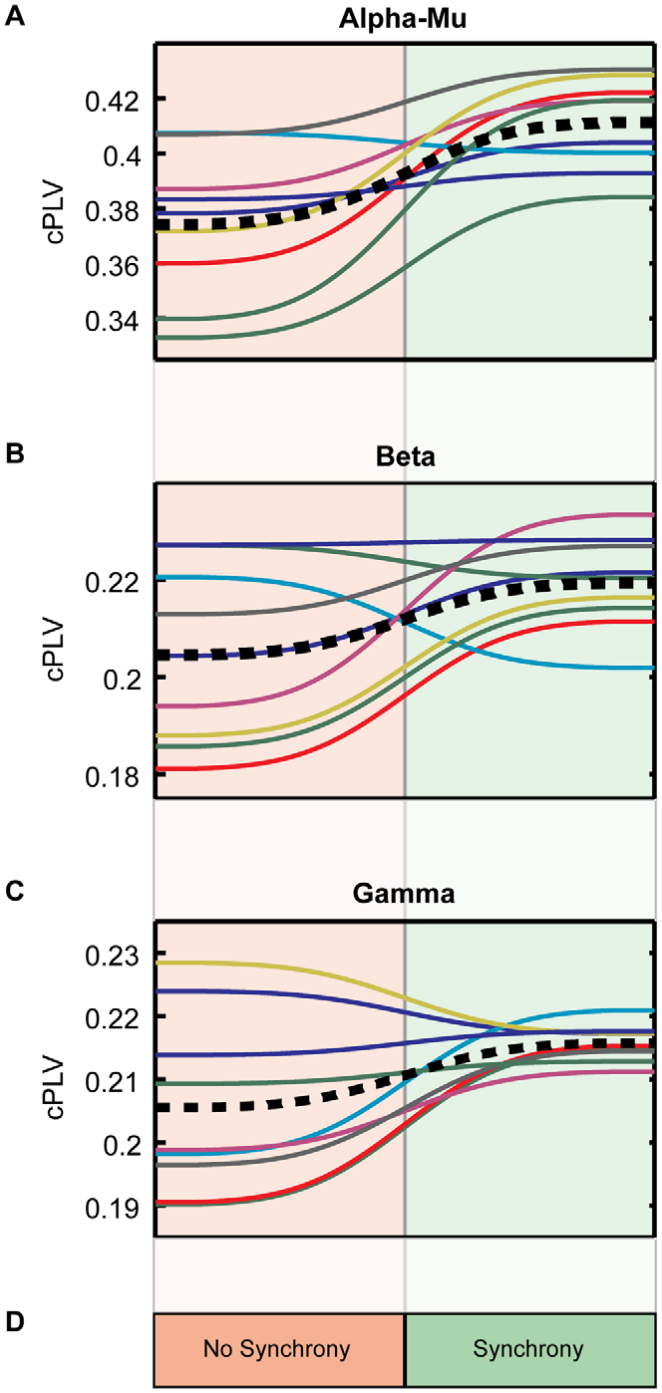
Summary of relevant intersubject synchronizations for all dyads according to interactional synchrony. cPLV values indicate the averaged PLVs on all pairs of electrodes members of clusters shown in Figure 2. Averages cPLV across dyads are shown in black dashed lines.

Figure 4 shows the mean PLV of all pairs within each significant cluster (cPLV) during Sync and NSync periods of SI. The global trends across all dyads confirmed that interbrain synchronization within our clusters corresponds to interactional synchrony.

**Figure 5:**
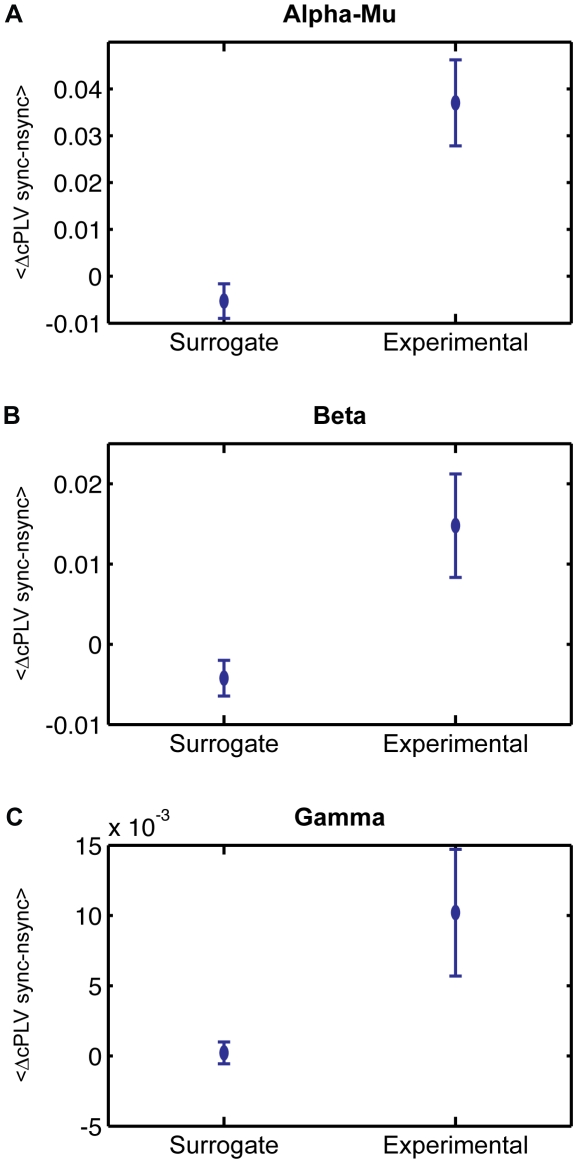
Averaged intersubject clustered PLV (cPLV) difference between synchronous and non-synchronous interactions (Sync - NSync) compared for experimental and surrogate behavioral analysis. Bars represent standard errors.

To test the validity of our experimental data, the PLV measure during episodes of behavioral Sync vs. Nsync was submitted to the technique of surrogate data. With this procedure, the timing between EEG data and behavioral data was broken by a shuffling of behavioral Sync and Nsync episodes. Accordingly, a surrogate PLV was obtained and compared to our experimental PLV data. Differences between the mean PLV over each cluster for *Sync vs. NSync* episodes were then computed using a Wilcoxon test. The analysis revealed that the PLV contrast was larger in the experimental than in the surrogate condition for the alpha-mu rhythm frequency (See Figure 5; T = 5, *p<0.05*).

#### Imitative versus Non Imitative episodes of SI condition

EEG contrasts performed between Im versus NIm episodes of the SI condition did not reveal significant differences for the distinct frequency bands whatever the scalp regions (CS<5.0, *p>0.05*).

#### Induced Imitation versus No View Motion

Significant EEG contrasts between II and NVM were found in the theta frequency bands only. The cluster statistics (CS) provided inter-frequency comparisons with the following absolute thresholds: 5.8, 10.5, 13.6 and 20.4 for respectively p<*0.05*, *p<0.01*, p<*0.005 and* p<*0.001*. Symmetrical increase in PLV was revealed between the right parieto-occipital regions of the model (CP2, P4, P8, PO10) and of the imitator (CP2, P3, PZ, PO2, POZ, PO10) in the theta frequency band (CS = +19.0, p<*0.005*).

## Discussion

To the best of our knowledge, this is the first study that recorded dual-EEG activity in dyads of subjects during spontaneous non verbal interaction. A few dual-EEG studies [14], [15], [20] have recently reported synchronous oscillations between brains in a social context. During true live interaction, our study provided evidence that behavioral synchrony and turn taking are accompanied by brain oscillatory couplings. Within one brain, synchronous neural oscillations have been previously observed in a range of processes such as conscious perception [44]–[46], working and long term memory [47], [48], states of anticipation or attention [48], [49] and empathy [50]. Such phase synchronizations have been proposed as a key mechanism for information integration [28], temporal binding [51], flexible neuronal communication [34], [52] and unified cognitive processes [45], [53]. Examining phase synchronizations between two brains, we discovered that they were related to several oscillatory frequency bands in the right centroparietal scalp regions of the two partners. The right temporoparietal region has been suggested to play a pivotal role in social interaction [31]. Previous fMRI studies indicated that the right temporoparietal region is consistently activated in both sociocognitive processes involving attention orientation, the sense of agency, self-other discrimination, perspective-taking [31], [32], and in the temporal analysis of visuomotor processing [54], [55]. Imitative interaction requires that participants share attention and compare cues arising from temporally distributed self and other's actions. Within a neurodynamic framework, the right parietal lobes of the two interactants could be seen as two functional hubs expected to synchronize during interaction.

During synchronous episodes, the emergence of a distributed functional network of interbrain neural synchronizations was found among several oscillatory bands. This is in line with current neurodynamic frameworks proposing that multiband synchronous oscillatory activity supports unified complex cognitive processes [45], [53], or serves as a mechanism for flexible and efficient communication among distinct or widely distributed cortical areas [56].

The comparison between synchronized and non synchronized acts showed statistical differences in interbrain phase synchronies for all frequency bands analyzed (alpha-mu, beta, and gamma) except for the theta band. Designing specific interbrain statistical analyses, we were able to show that the alpha-mu rhythm was the most robust interbrain oscillatory activity discriminating behavioral synchrony vs. non synchrony in the centroparietal regions of the two interacting partners. The alpha-mu band is considered as a neural correlate of the mirror neuron system functioning [57]. Specific frequencies of this band (9.2–11.5 Hz) over the right centroparietal region have been proposed as a neuromarker of social coordination [19]. The symmetrical pattern found for the model and the imitator possibly reflects a coordinated dynamics of hand movements. The pattern however became asymmetrical in the higher frequency bands and should be seen as a brain-to-brain top-down modulation reflecting the differential roles of model and imitator. This is consistent with motor transient activities involved in the beta band [57] and the implication of gamma in attentional processes, perceptual awareness and cognitive control [34], [58].

The other contrasts performed complement these findings. The absence of a significant difference between imitative and non-imitative episodes during spontaneous imitation assesses that interbrain synchronizations do not exclusively reflect the execution and perception of similar movements. By contrasting induced imitation with the NVM baseline no interbrain synchronization appeared, except in the theta band. The theta synchronization was found between the two right parietal regions but not in motor regions, although theta band is involved in the encoding of low level parameters of hand movements such as position [59]–[61] and speed [62]. Our finding could be explained by the fact that subjects move hands continuously in the two conditions, thus eliminating motor regions from the contrast. Right parietal locus could reflect a shift toward the processing of self-other similarities in the matched hand movements.

Overall, this study highlights the crucial and multiple roles of the right parietal regions in social interaction. Considering that subjects performed bimanual movements, the functional asymmetry between the two parietal areas is pointed out. What are the specificities of the right parietal regions? They have been considered as the “when pathway” [63], [64] because of its implication in the perception of time [65]. Beyond synchrony, alternation of roles involves temporal estimation and anticipation. Wilson & Wilson [6] have proposed that turn-taking, as a regulation of social interaction, should be supported by endogenous oscillators. The turn-taking phenomenon has been modeled in computer science and robotics studies [4], [5], [66] but never investigated so far in neuroscience. Here we show that interbrain neural synchronizations can be seen as reflecting in different bands several aspects of the ongoing social interaction, such as interactional synchrony, anticipation of other's actions and co-regulation of turn-taking. Although far more work is needed, the novel methodology used here offers a promising way to capture the brain to brain bases of the continuous flow of reciprocal influence that defines the core of social interaction.
